# Self-Perceived Impact of Oral Health on the Quality of Life of Women Deprived of Their Liberty

**DOI:** 10.1155/2021/5520652

**Published:** 2021-05-27

**Authors:** Ludmila Roberto Moraes, Lidiane Castro Duarte de Aquino, Danielle Teles da Cruz, Isabel Cristina Gonçalves Leite

**Affiliations:** ^1^Postgraduate Program in Collective Health, Universidade Federal de Juiz de Fora, Juiz de Fora, MG, Brazil; ^2^Department of Collective Health, School of Medicine, Universidade Federal de Juiz de Fora, Juiz de Fora, MG, Brazil

## Abstract

**Background:**

Prison units are marked by structural deficiencies, especially in relation to the female gender.

**Objectives:**

To measure the self-perceived impact of oral health on the quality of life of Brazilian women in detention. *Methodology*. A survey was carried out conducted in the penitentiary at Juiz de Fora (Minas Gerais, Brazil) using an instrument with semistructured questions and validated scales, including the Oral Health Impact Profile-14. 99 women were interviewed. The analysis was based on a theoretical model of determination, with hierarchical blocks of variables. Bivariate analysis was done using the Mann–Whitney, test and multivariate analysis was used using linear regression. The significance level was set at 5%.

**Results:**

33% experienced tooth loss after incarceration, (3.70 ± 3.26 lost teeth). 65.6% rated the dental service as fair/poor. The highest prevalence of oral health impact was for the domains of psychological discomfort (50.5%) and physical pain (40.4%). There is a negative impact on psychological discomfort: the number of dental consultations in the past year and self-perceived general health. There is an impact on physical pain: self-declared color and anxiety. Self-perceived general health had an impact on the domains of psychological disability and social disadvantage. Depression had an impact on the total score.

**Conclusion:**

This study revealed a self-perceived impact of oral health on the quality of life of women inmates. We need to ensure high-quality access to dental treatment in prisons.

## 1. Introduction

Brazil currently ranks fourth in countries with the largest female prison population in the world [1]. This population consists mainly of young women, black women, single women, and mothers, with low levels of education and economic status; female incarceration stands out for its high growth rate compared to the male group, and over 16 years (period from 2000 to 2016), this represented an increase of 656% nationally [2, 3].

Women deprived of their liberty are more affected by health problems than the general female population [4]. Among the vulnerabilities are those related to oral health, which can affect their perceived quality of life. The negative self-perception of health is related to indicators of social inequities manifesting the harmful action of the social determinants of health [5]. The impact of oral problems has been associated with such indicators in the same way that there is an association between the concept of quality of life and general aspects of health, including oral health [4–6].

Previous national research has demonstrated the severe shortage of health equipment within prison facilities. It stressed that the scarcity of social perspectives and access to health associated with prisonersʼ former lives carry on in prison life and concluded that prison institutions are marked by a set of structural deficiencies that prevent the state from ensuring that individualsʼ health is unscathed during their life in prison [4].

Recent studies on social deprivation measures have been correlated with oral health, providing data that confirm the validity of the concept presented by these measures, which indicates their usefulness in the formulation of oral health policies and decisions related to the allocation of resources to this area [7].

The Oral Health Impact Profile (OHIP) is an oral health-related quality of life instrument for adults that has demonstrated the importance and universality of the more general aspects of oral health. It is reported by the patient being characterized as a construct about the psychosocial and physical impact of oral disorders [7–11]. Considering self-perceived health as a relevant indicator and the context presented that treats the health of the female prison population as a public health problem, the present study was developed with the objective of measuring the self-perceived impact of oral health on the quality of life of this population and the associated factors in women detained in a prison unit in the city of Juiz de Fora-MG in Brazil.

## 2. Methods and Materials

This is a cross-sectional study, carried out using a census survey among women deprived of their liberty, over 18 years, detained for at least 30 days in the Eliane Betti womenʼs annex of the José Edson Cavalieri Penitentiary, located in the city of Juiz de Fora in the state of Minas Gerais. Juiz de Fora is the headquarters of the 4th Integrated Public Security Region, composed of four prison units and has the largest number of establishments and the largest concentration of inmates. According to the data from August 2020, around 2300 were maintained in the Integrated Prison Management System.

All the women detained in the provisional, closed, and semiopen regimes of the unit under study, included in the Integrated Prison Management System, from September 2019 to February 2020 were invited to participate. A total of 150 were women, of whom 51 were counted as losses: 21 resulted from a court order, 4 were due to transfer to another prison unit, and 26 women were not interviewed due to the interruption of the collection determined by the COVID-19 pandemic contingency measures in the prison resulting in 99 participants.

Data collection was preceded by a pilot study in another womenʼs prison unit, during which adjustments were made to the instrument and the form of application. The data collection instrument was composed of semistructured questions, developed from the instrument used in the research “Study of the Health Conditions and Quality of Life of Prisoners and the Environmental Conditions of Prison Units in the State of Rio de Janeiro” [4] and from scales validated for the Portuguese language. Symptoms of depression and anxiety were measured by PHQ-4 [12]. The study had as a dependent variable the Oral Health Impact Profile (OHIP-14), developed by Slade and Spencer, in 1994. The questionnaire used measures the limitation, discomfort, and disability attributed to the oral condition. It consists of two questions for each of the seven domains: functional limitation, physical pain, psychological discomfort, physical disability, psychological disability, social disability, and social disadvantage. The questions were organized in such a way that the inmates indicated, according to a Likert scale with five response categories, how often they experienced each problem, within a 6-month reference period. The response categories and respective values are as follows: always = 4; repeatedly = 3; sometimes = 2; rarely = 1; never = 0. For the descriptive analysis of the OHIP, the domain was considered as having an impact when self-perceived as repeatedly or always, with a minimum value of 3 and a maximum of 8; and without impact when self-perceived as never, rarely, or sometimes, receiving a value between 0 and 4 [8, 9, 13].

**Figure 1:**
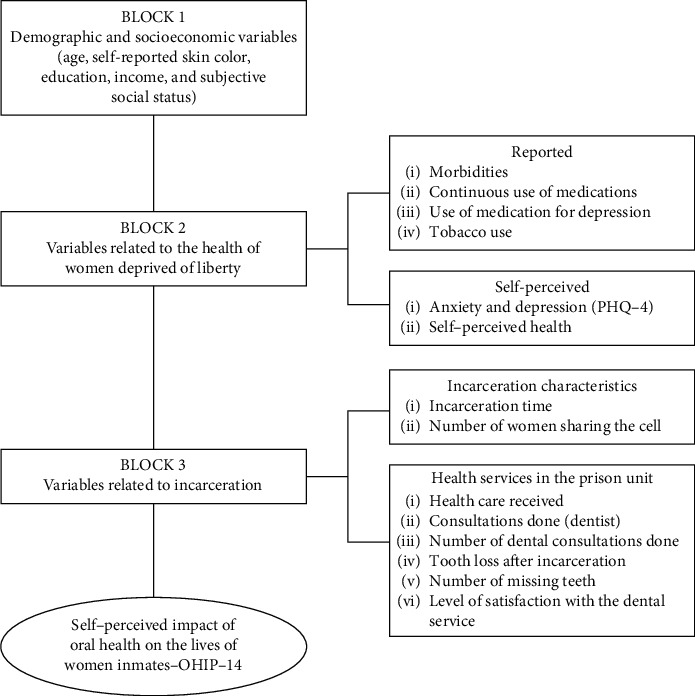
Theoretical model for investigating the effects of the independent variables on the self-perceived impact of oral health on the lives of women, in hierarchical blocks.

To analyze the factors associated with the self-perceived impact of oral health on womenʼs lives, a theoretical model of determination was constructed [14], illustrated in Figure 1, with three hierarchical blocks of independent variables.

Statistical analysis was performed using IBM SPSS software (version 15.0 for Windows, IBM Corp., Armonk, NY, USA) to obtain the absolute and relative frequency measures of the analyzed variables and central tendency and their dispersion measures. Normality was assessed using the Kolmogorov-Smirnov test. Bivariate analysis was conducted using a nonparametric Mann–Whitney test, given that the distribution pattern of the dependent variable in relation to the domains was asymmetric. As for the total OHIP, the distribution was normal on applying the *t*-test for independent samples. The variables with a significance level of ≤0.10 were included in the linear regression model and adjusted to the immediately higher level. In the multivariate linear regression analysis, the variables were controlled, adopting a significance level of 5%.

The present study is part of the macroproject entitled “Living and Health Conditions for Women Deprived of Liberty in Juiz de Fora-MG,” approved by the State Secretariat for Prison Administration and approved by the Research Ethics Committee of the Federal University of Juiz de Fora, in the form of Opinion no. 3.294.253.

## 3. Results

The population of this study (*n* = 99) was composed of women with a mean age of 33 years (±9.2), mostly nonwhite (77.8%), having up to 9 years of schooling (76.7%), and income up to $183.00 (Brazilian minimum wage) before incarceration (66.7%). As for their subjective social status, 81.9% of the inmates considered themselves at the lowest level according to the MacArthur subjective social status scale (Table 1). Regarding the incarceration characteristics, the mean time of incarceration of 25 months (±38.01) and the average number of women per ell of 18.17 (±4.37) (Table 1) are highlighted.

Regarding the health care received, 90.9% responded that they received care, with the lowest percentage of assistance reported by the inmates being related to dental care (58.6%). Thirty-three percent (33%) of the inmates said they had experienced tooth loss after incarceration, with a mean of 3.70 (±3.26) teeth lost. Along this line, 65.6% rated the level of satisfaction with the dental service as fair or poor (Table 2).

Most of the inmates classified their health as good (58.6%), although 52.5% reported having some health problems. They make continuous use of medications (70.7%), with 33.3% for depression. In all, 72.7% smoke, with a mean of 15.68 years of smoking (±10.09). A significant number of women reported some symptoms of anxiety and/or depression (86.9%) (Table 3).

**Figure 2:**
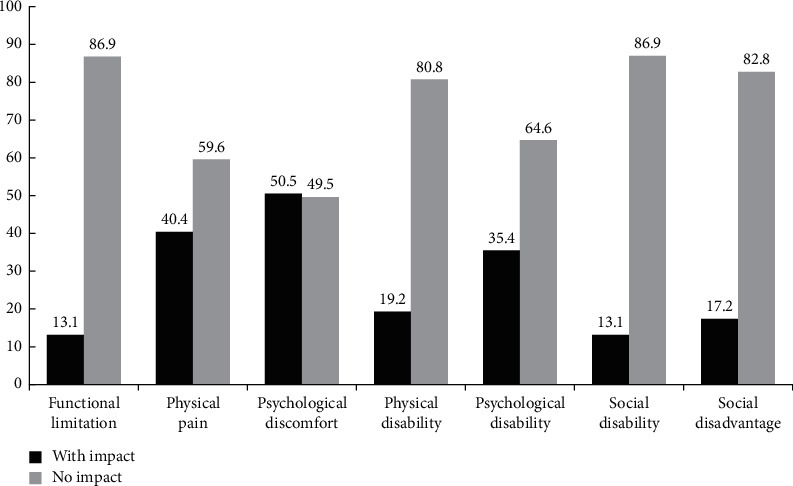
Distribution of women inmates in Juiz de Fora, according to the prevalence of impact, by OHIP-14 domains, Juiz de Fora, 2020.

Figure 2 shows the prevalence of impact for each OHIP domain. The domains physical pain (40.4%) and psychological discomfort (50.5%) presented the highest prevalence of oral health impact.

Table 4 shows the comparison of strata of the independent variables by domain and final OHIP-14 score (Table 4).

Table 4 shows the result of the multiple linear regression analysis. Regarding the physical pain domain, the variables that remained significant after adjustment were self-declared color and anxiety symptoms. These variables explain 26% of the variability of this domain. For the psychological discomfort domain, after adjustment, the variables that remained significant were the number of dental consultations in the past year and self-perceived general health, which explain almost 35% of the variability of this domain. For the psychological disability and social disadvantage domains, there was a significant association with self-perceived general health, which explains 24% and 25% of the variability of those domains, respectively (Table 5).

## 4. Discussion

This study sought to measure the self-perceived impact of oral health on the quality of life of women deprived of liberty in a prison unit in Minas Gerais. The domains with the highest prevalence of impact for the studied population were psychological discomfort (50.05%), physical pain (40.4%), psychological disability (35.4%), and social disadvantage (17.2%). The following variables remained associated with the outcome of psychological discomfort in the final model: the number of dental consultations in the past year (*p*=0.015) and self-perceived general health (*p*=0.024); for the physical pain outcome, the associated variables were self-reported color (*p*=0.021) and anxiety (*p*=0.046); as for the outcomes psychological disability and social disadvantage, they were associated with the variable self-perceived general health (*p*=0.010 and *p*=0.011, respectively). The total OHIP-14 presented an association with the depression variable (*p*=0.011).

The study reported that 77.8% of the interviewees declared themselves to be nonwhite, the same profile found in a survey with inmates of a penitentiary in São Paulo [15] and by the survey by DEPEN (National Penitentiary Department) [2]. In the present study, the self-declared nonwhite color variable was associated with a worse result for the physical pain domain, which, according to Slade [9], is the domain that presents impacts of caries and periodontal diseases. This result is in line with other studies with the same population profile in different regions of the country [16–18]. Along the same lines, a study carried out in a city in the state of Minas Gerais with different profile groups demonstrated that less access to oral health services is experienced by brown women (68%) who had greater impacts of physical pain on their quality of life [19]. Another study indicates that, in the specific case of dental care, the percentage of blacks who never went to the dentist reaches 24%, while the percentage of whites with a similar lack of access reaches 14% [20].

The history of dental disease caries and periodontal disease seems to be a frequent finding that affects the quality of life of incarcerated populations, as revealed by research in India [21]. Oral diseases can be considered a biological expression of social factors. A study among socially deprived people reports more negative perceptions and behaviors regarding health compared to the socially affluent [22].

There are few studies in the literature with a female prison population in custody that have verified the impact of oral health using the OHIP as the instrument, which makes it difficult to compare the present findings. The study obtained a total OHIP mean of 15.13, which differs from a study carried out among workers from a Brazilian federal university, considering that the total OHIP-14 mean was 4.55, in which the majority had a graduate school education. This indicates that education level reflects aspects related to access to health services [23] although, in the present study, the variable did not prove significant for the outcome.

Family income of less than 1 x minimum wage, as was the case for 66.6% of these women inmates, showed a significant association (*p*=0.049) in the bivariate analysis for the functional limitation domain. The oral health inequities can be determined by exclusionary social contexts, which increase the vulnerability of population groups [20]. An Indian study, in an industrial environment with adults, found that individuals with a higher socioeconomic status had fewer decayed and missing teeth and less frequent impacts on their daily lives, that is, better oral health-related quality of life, which demonstrates the socioeconomic stratification of oral health results and the social determination in health [24].

As for the variable, the number of dental consultations in the past year, 58.6% of the inmates received only 1 dental consultation, being unfavorably associated with the psychological discomfort domain, which means a hindrance to performance activities, such as eating [9]. In India, the high prevalence of oral morbidities among prisoners can be attributed to the lack of dental services offered by the penitentiary system [21]. A very similar situation takes place in the prison unit studied here, which, although it has a dental office, is not concerned with real dental service provision, serving only as a screening service within the Inclusion and Resocialization Program, which results in an assessment of satisfaction with service as fair or poor (65.6%) since it is not able to resolve issues related to the oral health problems of that population.

The severity of oral health impact on quality of life may be associated with the limited access to dental services. Cohen-Carneiro et al. [25] found high means for the total OHIP-14 for two riverside communities in the Amazon (10.92 and 14.03). The authors justified that these results were explained by the limited access to dental services in this region, due to the distance from urban centers. A Scottish study carried out in three prisons to verify the factors associated with access to dental service found a mean OHIP-14 score of 15.61, very close to that found in the present study, demonstrating the importance of this association for the oral health impact on the quality of life for populations deprived of their liberty [25].

Regarding self-perceived general health, 68.7% of the inmates reported it being very good or good. Conversely, 61.5% of the women in detention in São Paulo rated their health negatively [18]. Gabardo et al. [5] show that negative self-perceived oral health is related to indicators of social inequities, such as low income and low education.

As for the mental health of the study population, 63.6% of the inmates had positive symptoms of anxiety, while 42.4% had positive symptoms for depression, according to the PHQ-4 subscales. The high prevalence of such symptoms observed in our study population is consistent with a study with incarcerated individuals in Rio de Janeiro, and both reflect national and international data that ratify the concern with these mental disorders among prisoners, especially women [26–28].

An American study indicated that around 70% of the female prisoners had at least one mental health problem [29]. Another study in a prison unit in that same country revealed that more than half of the detainees had positive symptoms for anxiety and that over two-thirds of the detainees would potentially develop some psychiatric disorders throughout their lives, finding, lastly, that psychiatric morbidity estimates in women were higher than in men, data which reiterate that the provision of mental health services in prisons is crucial, especially care for the female sex [30].

The entire complexity of issues related to confinement exposes women to a convergence of feelings such as pain, sadness, abandonment due to the interruption of family relationships, loneliness, stress, anxiety, and depression that negatively affect health and life conditions, as determined by Araújo et al. [31] and Santos et al. [2]. These converge with the results of this study, since symptoms of anxiety and depression proved to be associated with OHIP domains and, therefore, constitute important factors associated with oral health impact on the inmatesʼ quality of life.

In the present study, the anxiety variable was significantly associated with the physical pain domain, while the depression variable was significantly associated with total OHIP. Locker and Quiñonez [32], in a study among the elderly, found that mental health impairment on quality of life and a higher OHIP score were directly proportional. Therefore, the impacts of oral disorders in their physical and psychosocial domains were associated with a worse quality of life, when there was mental health impairment.

The data collected in the study by Arora et al. [33] in prisons in Scotland demonstrated the comorbidity relationship between mental health and oral health and demonstrated the importance of incorporating oral health policy into prison and inclusion of oral health promotion measures to ensure that these needs are more adequately met, reducing impacts on the quality of life of these prisoners.

The presence of people with mental health problems in prisons in the United States was identified three times more frequently than in US hospitals [33]. Also in this regard, a study carried out in Scottish prisons found that the prevalence of depressed individuals in these environments is double that in the Scottish population [32]. Studies in prisons in Paraíba, São Paulo, and Rio de Janeiro revealed a high prevalence of mental disorders in the female prison population [2, 34–36].

Constantino et al. [37], in a study carried out in a prison unit in the state of Rio de Janeiro, and Damas and Oliveira [29], in a study carried out in a prison unit in Santa Catarina, reinforce that the findings of the mental health impairment of the incarcerated population are related not only to experiences prior to entering the prison system but to the conditions inherent in incarceration. This situation contradicts international principles that impose a responsible and humanized custody posture on prisoners so that they do not leave prison in a worse state of health than when they entered it [37].

In sum, various studies indicate that issues related to incarceration, socioeconomic, demographic, mental health, and general health conditions have an impact on the quality of life of the female population deprived of their liberty. The study presented a limitation attributed to the lack of clinical data on the oral health of women inmates, since conducting dental examinations would require rigorous logistical management of inmates by penal officers, a flow that was not facilitated by the prison unit under study. Data on oral health conditions were self-reported. However, the study innovates by measuring the oral health impact on the quality of life of the female population in custody in different domains, being physical, social, and psychological.

## 5. Conclusion

Our study revealed the self-perceived impact of oral health on the quality of life of women deprived of their liberty in a prison unit in Minas Gerais. These results serve to indicate the need for greater investment in the prison system, in the sense of expanding and qualifying oral health services in order to allow access to vulnerable reeducates, and it is also expected that the findings of this research may support health strategies for women: incarcerated women not only in the municipality of Juiz de Fora but also for the entire female prison population.

The study served to indicate the need for greater investment in the prison system, in the sense of expanding and qualifying oral health services in order to allow access for women in custody in situations of vulnerability.

## Figures and Tables

**Table 1:** Demographic and socioeconomic characteristics of women inmates in Juiz de Fora, 2020.

Variables	Absolute frequency (*n*)	Relative frequency (%)
*Demographic characteristics*		
Age (in years)		
20–29	36	36.4
30–39	39	39.3
40–49	17	17.2
50–59	7	7.1

Self-reported skin color		
White	22	22.2
Black	33	33.3
Brown	41	41.5
Yellow/indigenous	3	3.0

*Socioeconomic characteristics*		
Schooling (in years)		
Illiterate	1	1.0
≥1 and <9	60	60.5
9	16	16.2
>9 and <12	5	5.1
12	15	15.2
Complete higher education	2	2.0

Profession		
Service workers, retail salespeople in stores and markets	61	61.6
Unemployed	9	9.1
Unpaid (student and at home)	7	7.1
Others	22	22.2

Family income (*x* minimum wage)		
0 to ≤ 0.5	26	26.3
>0.5 and ≤1.0	40	40.3
>1.0 and ≤1.5	16	16.2
>1.5 and ≤2.0	10	10.1
>2.0	7	7.1

MacArthur Scale of Subjective Social Status		
Very poor (1 and 2)	28	28.3
Poor (3 and 4)	25	25.2
Fair (5 and 6)	28	28.2
Good (7 and 8)	12	12.2
Very good (9 and 10)	6	6.1

**Table 2:** Incarceration characteristics and use of health services in the prison unit, women inmates in Juiz de Fora, 2020.

Variables	Absolute frequency (*n*)	Relative frequency (%)
*Incarceration characteristics*		
Reason for a prison sentence		
Crime against a person	12	12.1
Crime against property	30	30.3
Drug trafficking	52	52.5
Others	5	5.1

Imprisonment time (in months)		
≥1 and ≤12	45	45.4
>12 and ≤48	41	41.4
>48 and ≤96	8	8.1
>96	5	5.1

Sentence received (in years)		
Up to 4	9	12.8
>4 and ≤8	27	38.6
>8	30	42.9
Did not have an answer	4	5.7

How many women share the cell?		
10–15	27	27.3
16–20	26	26.2
>20	46	46.5
Mean (SD) 18.17 (4.37)		

Health services in the prison unit		
Health care received		
Yes	90	90.9
Consultations done with dentist		
Yes	58	58.6
No. of dental consultations in the past year		
1	34	58.6
2	15	25.9
3	3	5.2
4	6	10.3
Tooth loss after incarceration		
Yes	33	33.3
Number of teeth lost after incarceration		
1	9	27.3
>1 and ≤4	16	48.5
>4	8	24.2
Mean (SD) 3.70 (3.26)		
Level of satisfaction with dental service		
Poor	24	37.5
Fair	18	28.1
Good	22	34.4

**Table 3:** Characterization of women inmates in Juiz de Fora, according to self-perceived health and morbidity reported, Juiz de Fora, 2020.

Variables	Absolute frequency (*n*)	Relative frequency (%)
Self-perceived health		
Very good	10	10.1
Good	58	58.6
Fair	23	23.2
Poor	5	5.1
Very poor	3	3.0

Morbidity reported		
Yes	52	52.5

Continuous use of medications		
Yes	70	70.7

Medication for depression		
Yes	33	33.3

Tobacco use		
Yes	72	72.7

Symptoms of anxiety and depression		
None	13	13.1
Mild	19	19.2
Moderate	24	24.2
Serious	43	43.5

Anxiety (PHQ-4)		
Negative (<3)	36	36.4
Positive (≥3)	63	63.6

Depression (PHQ-4)		
Negative (<3)	57	57.6
Positive (≥3)	42	42.4

**Table 4:** Mean, standard deviation, and *p* value^†^ of the independent variables, by domains and for total OHIP-14 of women inmates in Juiz de Fora, 2020.

Variable	*n*	Means by domain (SD)							
		1	2	3	4	5	6	7	Total OHIP
Age (in years)									
≤33	61	1.33 (1.91)	3.69 (2.85)	3.61 (2.83)	1.44 (2.08)	2.36 (2.59)	1.21 (1.94)	1.25 (2.20)	15.07 (12.64)
>33	38	1.58 (2.35)	3.21 (2.48)	3.68 (2.88)	2.05 (2.75)	2.71 (2.25)	1.05 (1.64)	1.29 (2.10)	15.24 (11.79)
*p* value		0.852	0.455	0.933	0.435	0.283	0.871	0.746	0.575

Self-reported skin color									
White	22	0.64 (1.25)	2.45 (2.02)	3.23 (3.12)	2.00 (2.54)	2.82 (2.54)	0.86 (1.49)	0.68 (1.21)	12.05 (9.87)
Nonwhite	77	1.65 (2.22)	3.81 (2.82)	3.75 (2.76)	1.58 (2.32)	2.40 (2.45)	1.23 (1.91)	1.43 (2.34)	16.01 (12.78)
*p* value		0.031	0.052	0.393	0.287	0.428	0.476	0.287	0.173

Schooling (in years completed)									
<9	61	1.57 (2.31)	3.34 (2.70)	3.36 (2.80)	1.41 (2.12)	2.36 (2.45)	1.07 (1.92)	1.23 (2.21)	14.67 (12.51)
≥9	38	1.18 (1.66)	3.76 (2.74)	4.08 (2.87)	2.11 (2.69)	2.71 (2.49)	1.29 (1.67)	1.32 (2.09)	15.87 (11.98)
*p* value		0.748	0.455	0.221	0.261	0.515	0.206	0.695	0.334

Family income (*x* minimum wage)^‡^									
<1	66	1.17 (1.96)	3.47 (2.65)	3.73 (2.86)	1.44 (2.20)	2.56 (2.63)	1.17 (1.90)	1.35 (2.19)	14.92 (12.46)
≥1	33	1.94 (2.26)	3.58 (2.87)	3.45 (2.83)	2.15 (2.65)	2.36 (2.12)	1.12 (1.69)	1.09 (2.10)	15.55 (12.04)
*p* value		0.049	0.928	0.611	0.162	0.902	0.983	0.670	0.956

MacArthur Scale of Subjective Social Status^§^									
Poor	53	1.58 (2.15)	3.81 (2.86)	3.98 (2.78)	1.64 (2.39)	2.62 (2.47)	1.34 (2.00)	1.43 (2.22)	16.75 (13.08)
Good	46	1.24 (2.01)	3.15 (2.52)	3.24 (2.88)	1.72 (2.36)	2.35 (2.47)	0.93 (1.60)	1.07 (2.08)	13.26 (11.09)
*p* value		0.399	0.275	0.174	0.631	0.530	0.239	0.266	0.443

Imprisonment time (in months)									
≤15	52	1.54 (2.26)	3.90 (2.95)	3.85 (3.06)	1.48 (2.32)	2.58 (2.67)	1.21 (1.94)	1.44 (2.53)	16.48 (13.32)
>15	17	1.30 (1.89)	3.06 (2.38)	3.40 (2.58)	1.89 (2.42)	2.40 (2.22)	1.09 (1.70)	1.06 (1.65)	13.64 (10.92)
*p* value		0.769	0.177	0.501	0.221	0.934	0.805	0.987	0.141

How many women share the cell?									
≤17	53	1.72 (2.27)	3.60 (2.94)	3.58 (2.94)	1.81 (2.64)	2.32 (2.37)	1.08 (1.74)	1.51 (2.55)	15.98 (13.34)
>17	46	1.09 (1.81)	3.39 (2.44)	3.70 (2.74)	1.52 (2.02)	2.70 (2.57)	1.24 (1.93)	0.98 (1.56)	14.15 (10.95)
*p* value		0.097	0.859	0.811	0.982	0.518	0.602	0.865	0.790

Health care received									
Yes	90	1.41 (2.06)	3.34 (2.74)	3.62 (2.86)	1.71 (2.43)	2.44 (2.38)	1.13 (1.81)	1.28 (2.19)	14.84 (12.01)
No	9	1.56 (2.45)	5.11 (1.83)	3.78 (2.77)	1.33 (1.73)	3.00 (3.28)	1.33 (2.06)	1.11 (1.83)	18.00 (15.05)
*p* value		0.973	0.046	0.829	0.857	0.782	0.715	0.948	0.465

Dental consultation done									
Yes	58	1.31 (1.88)	3.79 (2.94)	4.10 (2.96)	1.93 (2.67)	2.62 (2.50)	1.31 (1.82)	1.24 (2.10)	16.26 (12.64)
No	41	1.59 (2.35)	3.10 (2.33)	2.98 (2.54)	1.32 (1.84)	2.32 (2.42)	0.93 (1.84)	1.29 (2.25)	13.54 (11.67)
*p* value		0.842	0.293	0.065	0.578	0.537	0.159	0.840	0.521

No. of dental consultations in the past year									
1	34	1.00 (1.58)	3.09 (3.02)	3.21 (3.00)	2.29 (2.83)	2.18 (2.82)	0.91 (1.76)	0.74 (1.52)	12.50 (12.31)
>1	24	1.75 (2.21)	4.79 (2.55)	5.38 (2.43)	1.42 (2.37)	3.25 (1.82)	1.88 (1.78)	1.96 (2.60)	21.58 (11.32)
*p* value		0.170	0.018	0.006	0.163	0.025	0.013	0.039	0.720

Tooth loss after incarceration									
Yes	33	1.64 (2.12)	4.21 (2.55)	4.58 (2.47)	1.82 (2.58)	3.21 (2.22)	1.39 (1.95)	1.88 (2.34)	19.42 (11.89)
No	66	1.32 (2.08)	3.15 (2.74)	3.17 (2.91)	1.61 (2.27)	2.14 (2.51)	1.03 (1.76)	0.95 (2.00)	12.98 (11.96)
*p* value		0.439	0.051	0.014	0.848	0.018	0.355	0.012	0.892

Number of teeth lost after incarceration									
<4	21	1.76 (2.30)	4.48 (2.56)	4.90 (2.23)	1.71 (2.81)	3.52 (2.23)	1.71 (1.98)	2.10 (2.51)	21.43 (11.98)
≥4	12	1.42 (1.83)	3.75 (2.56)	4.00 (2.86)	2.00 (2.22)	2.67 (2.19)	0.83 (1.85)	1.50 (2.07)	15.92 (11.38)
*p* value		0.855	0.496	0.471	0.425	0.456	0.145	0.647	0.593

Level of satisfaction with dental service									
Poor	77	1.58 (2.21)	3.58 (2.70)	3.60 (2.82)	1.62 (2.34)	2.60 (2.48)	1.23 (1.92)	1.43 (2.22)	15.81 (12.69)
Good	22	0.86 (1.46)	3.23 (2.81)	3.77 (2.97)	1.86 (2.49)	2.14 (2.40)	0.86 (1.42)	0.68 (1.84)	12.77 (10.57)
*p* value		0.280	0.537	0.827	0.638	0.443	0.634	0.075	0.195

Self-perceived health									
Good	68	1.13 (1.98)	3.16 (2.71)	3.15 (2.65)	1.65 (2.29)	1.99 (2.18)	1.06 (1.66)	0.78 (1.77)	12.72 (11.31)
Poor	31	2.06 (2.19)	4.26 (2.61)	4.71 (2.98)	1.74 (2.57)	3.61 (2.69)	1.35 (2.15)	2.32 (2.55)	20.42 (12.79)
*p* value		0.014	0.054	0.014	0.987	0.004	0.699	<0.001	0.324

Morbidity reported									
Yes	52	1.56 (1.97)	3.40 (2.54)	3.71 (2.86)	1.67 (2.29)	2.73 (2.51)	1.29 (1.86)	1.35 (1.92)	15.46 (11.62)
No	47	1.28 (2.21)	3.62 (2.92)	3.55 (2.84)	1.68 (2.48)	2.23 (2.41)	1.00 (1.79)	1.17 (2.41)	14.77 (13.05)
*p* value		0.127	0.790	0.798	0.884	0.286	0.229	0.243	0.294

Continuous use of medications									
Yes	72	1.38 (1.97)	3.58 (2.68)	3.69 (2.80)	1.56 (2.13)	2.60 (2.41)	1.18 (1.80)	1.33 (2.23)	15.33 (11.74)
No	27	1.56 (2.39)	3.30 (2.83)	3.48 (2.99)	2.00 (2.92)	2.22 (2.62)	1.07 (1.80)	1.07 (1.96)	14.59 (13.77)
*p* value		0.762	0.599	0.702	0.676	0.384	0.581	0.749	0.239

Medication for depression									
Yes	33	1.52 (2.28)	4.09 (2.38)	3.91 (2.83)	1.61 (2.28)	3.03 (2.35)	1.06 (1.58)	1.76 (2.51)	17.21 (11.27)
No	66	1.38 (2.00)	3.21 (2.84)	3.50 (2.85)	1.71 (2.43)	2.23 (2.49)	1.20 (1.95)	1.02 (1.92)	14.09 (12.68)
*p* value		0.886	0.081	0.469	0.938	0.077	0.814	0.097	0.349

Tobacco use									
Yes	72	1.56 (2.21)	3.94 (2.70)	4.10 (2.92)	1.54 (2.34)	2.71 (2.51)	1.28 (1.99)	1.56 (2.38)	16.99 (12.70)
No	22	1.07 (1.68)	2.33 (2.42)	2.41 (2.22)	2.04 (2.44)	1.93 (2.25)	0.81 (1.24)	0.48 (1.09)	10.19 (9.56)
*p* value		0.379	0.008	0.011	0.244	0.167	0.599	0.030	0.173

Anxiety symptoms									
Negative	36	0.89 (1.74)	2.69 (2.45)	2.97 (2.35)	1.78 (2.63)	1.97 (2.31)	1.17 (1.68)	0.78 (1.55)	11.64 (10.19)
Positive	63	1.74 (2.22)	3.97 (2.77)	4.02 (3.03)	1.62 (2.22)	2.79 (2.51)	1.14 (1.92)	1.54 (2.40)	17.13 (12.96)
*p* value		0.041	0.025	0.098	0.997	0.099	0.639	0.116	0.164

Depression symptoms									
Negative	57	1.05 (1.74)	3.47 (2.65)	3.12 (2.50)	1.65 (2.40)	2.00 (2.20)	0.95 (1.53)	0.63 (1.29)	12.46 (10.24)
Positive	42	1.93 (2.41)	3.55 (2.83)	4.33 (3.14)	1.71 (2.35)	3.17 (2.65)	1.43 (2.15)	2.12 (2.74)	18.76 (13.88)
*p* value		0.066	0.957	0.048	0.658	0.026	0.396	0.002	0.003

†The Mann–Whitney test was used to calculate the *p* value of the OHIP-14 domains. For the *p* value of the total OHIP-14 score, the *t*-test of independent samples was used. Statistically significant (*p* < 0.05). ‡The current national minimum wage in the amount of R$ 1,045.00 (one thousand and forty-five reais), which corresponds to U$ 187 (one hundred and eighty-seven dollars). §Continuous variable with a score ranging from 1 to 10 and dichotomized into poor and good representing, respectively, the categories: “very poor” and “poor” (steps 1 and 2; 3 and 4), and “fair,” “good,” and “very good” (5 and 6; 7 and 8; 9 and 10).

**Table 5:** Multiple linear regression model: factors associated with OHIP for women inmates in Juiz de Fora, 2020.

	Physical pain			Psychological discomfort^*∗∗*^			Psychological disability			Social disadvantage			OHIP-14		
Adjusted *r*^2^	0.262			0.345			0.239			0.255			0.056		
Variables	*β*	95% CI	*p*	*β*	95% CI	*p*	*β*	95% CI	*p*	*β*	95% CI	*p*	*β*	95% CI	*p*
Self-declared color (white)	1.84	0.29; 3.38	0.021												
No. of dental consultations in the past year (1)	0.62	−0.14; 1.39	0.109	0.88	0.18; 1.59	0.015	0.25	−0.39; 0.89	0.438	0.21	−0.33; 0.76	0.441			
Self-perceived general health (good)	1.15	−0.46; 2.76	0.158	1.88	0.26; 3.51	0.024	1.94	0.48; 3.41	0.010	1.62	0.39; 2.85	0.011			
Anxiety symptoms (negative)	1.52	0.02; 3.02	0.046	0.84	−0.80; 2.47	0.305	0.64	−0.84; 2.11	0.390						
Depression symptoms (negative)				1.25	−0.43; 2.93	0.142	0.64	−0.89; 2.17	0.404	1.07	−0.04; 2.17	0.058	6.31	1.50; 11.12	0.011

^*∗∗*^The variable consultation done at the dentist was excluded for autocorrelation.

## Data Availability

The SPSS file (*∗*.sav) data used to support the findings of this study are available from the corresponding author upon request.

## References

[1] Institute for Crime & Justice Policy Research (2020). *About World Prison Brief*.

[2] Santos T., Rosa M. I., Devoti H. R. F. (2017). *Levantamento Nacional de Informações Penitenciárias Infopen Mulheres*.

[3] Lima G. M. B., Pereira Neto A. F., Amarante P. D. C. (2013). Mulheres no cárcere: significados e práticas cotidianas de enfrentamento com ênfase na resiliência. *Saúde Debate*.

[4] Minayo M. C. S., Constantino P. (2015). *Deserdados Sociais: Condições de Vida e Saúde dos Presos do Estado do Rio de Janeiro*.

[5] Gabardo M. C. L., Moysés S. T., Moysés S. J. (2013). Autopercepção de saúde bucal conforme o Perfil de Impacto da Saúde Bucal (OHIP) e fatores associados: revisão sistemática. *Revista Panamericana de Salud Pública*.

[6] Minayo M. C. S., Hartz Z. M. A., Buss P. M. (2000). Qualidade de vida e saúde: um debate necessário. *Ciência & Saúde Coletiva*.

[7] Locker D. (1988). Measuring oral health: a conceptual framework. *Community Dental Health*.

[8] John M. T., Reißmann D. R., Feuerstahler L. (2014). Factor analyses of the oral health impact profile: overview and studied population. *Journal of Prosthodontic Research*.

[9] Slade G. D. (1997). Derivation and validation of a short-form oral health impact profile. *Community Dentistry and Oral Epidemiology*.

[10] Oliveira B. H., Nadanovsky P. (2005). Psychometric properties of the Brazilian version of the oral health impact profile-short form. *Community Dentistry and Oral Epidemiology*.

[11] Vettore M. V., Aqeeli A. (2016). The roles of contextual and individual social determinants of oral health-related quality of life in Brazilian adults. *Quality of Life Research*.

[12] Löwe B., Wahl I., Rose M. (2010). A 4-item measure of depression and anxiety: validation and standardization of the Patient Health Questionnaire-4 (PHQ-4) in the general population. *Journal of Affective Disorders*.

[13] Jokstad A. (2018). Editorial. Patient-reported outcomes (PROs) versus patient-reported outcome measures (PROMs)-Is there a difference?. *Clinical and Experimental Dental Research*.

[14] Victora C. G., Huttly S. R., Fuchs S. C. (1997). The role of conceptual frameworks in epidemiological analysis: a hierarchical approach. *International Journal of Epidemiology*.

[15] Audi C. A. F., Santiago S. M., Andrade M. da G. G. (2016). Inquérito sobre condições de saúde de mulheres encarceradas. *Saúde Debate*.

[16] Soares B. M., Ilgenfritz I. (2002). *Prisioneiras: Vida e Violência Atrás das Grades*.

[17] Rodrigues I. S. A. A., Silveira I. T. de M., Pinto de M. S. A. (2014). Locked mouths: tooth loss in a women’s prison in Northeastern Brazil. *The Scientific World Journal*.

[18] Bezerra R. de C. C., Fernandes R. A. Q. (2015). Perfil social e de saúde de mulheres apenadas de uma penitenciária da cidade de São Paulo. *Perspectivas Médicas*.

[19] Pinho A. M. S., Campos A. C., Ferreira E. (2012). Toothaches in the daily lives of Brazilian adults. *International Journal of Environmental Research and Public Health*.

[20] Guiotoku S. K., Moysés S. T., Moysés S. J. (2012). Iniquidades raciais em saúde bucal no Brasil. *Revista Panamericana de Salud Pública*.

[21] Fotedar S., Chauhan A., Bhardwaj V. (2016). Association between oral health status and oral health-related quality of life among the prison inmate population of kanda model jail, Shimla, Himachal Pradesh, India. *Indian Journal of Public Health*.

[22] Locker D. (2000). Deprivation and oral health: a review. *Community Dentistry and Oral Epidemiology*.

[23] Guerra M. J. C., Greco R. M., Leite I. C. G. (2014). Impact of oral health conditions on the quality of life of workers. *Ciência & Saúde Coletiva*.

[24] Gupta E., Robinson P. G., Marya C. M. (2015). Oral health inequalities: relationships between environmental and individual factors. *Journal of Dental Research*.

[25] Cohen-Carneiro F., Souza-Santos R., Rebelo M. A. B. (2011). Quality of life related to oral health: contribution from social factors. *Ciência & Saúde Coletiva*.

[26] Freeman R., Richards D. (2019). Factors associated with accessing prison dental services in Scotland: a cross-sectional study. *Journal of Dentistry*.

[27] Kravitz H. M., Cavanaugh J. L., Rigsbee S. S. (2002). A cross-sectional study of psychosocial and criminal factors associated with arrest in mentally ill female detainees. *Journal of the American Academy of Psychiatry and the Law*.

[28] Silva N. C., Rosa M. I., Amboni G. (2011). Transtornos psiquiátricos e fatores de risco em uma população carcerária Psychiatric disorders and risk factors in a prison population. *Arquivos Catarinenses de Medicina*.

[29] Damas F. B., Oliveira W. F. (2013). A saúde mental nas prisões de Santa Catarina, Brasil. *Brazilian Journal of Mental Health*.

[30] James D. J., Glaze L. E. (2006). *Mental Health Problems of Prison and Jail Inmates*.

[31] Araújo de M. M., Moreira da A. S., Cavalcante E. G. R. (2020). Health care for incarcerated women: analysis based on the theory of basic human needs. *Escola Anna Nery*.

[32] Locker D., Quiñonez C. (2011). To what extent do oral disorders compromise the quality of life?. *Community Dentistry and Oral Epidemiology*.

[33] Arora G., Humphris G., Lahti S. (2020). Depression, drugs and dental anxiety in prisons: a mediation model explaining dental decay experience. *Community Dentistry and Oral Epidemiology*.

[34] Cloud D., Dougherty M., May R. L. (2014). At the intersection of health and justice. *Perspectives in Health Information Management*.

[35] Oliveira L. V., Costa G. M. C., Medeiros K. K. A. S. (2013). Epidemiological profile of female detainees in the Brazilian state of Paraíba: a descriptive study. *Online Brazilian Journal of Nursing*.

[36] Andreoli S. B., dos Santos M. M., Quintana M. I. (2014). Prevalence of mental disorders among prisoners in the state of Sao Paulo, Brazil. *PLoS One*.

[37] Constantino P., Assis de S. G., Pinto L. W. (2016). O impacto da prisão na saúde mental dos presos do estado do Rio de Janeiro, Brasil. *Ciência & Saúde Coletiva*.

